# A Real‐World Multicentre Retrospective Study of Paclitaxel‐Bevacizumab and Maintenance Therapy as First‐Line for HER2‐Negative Metastatic Breast Cancer

**DOI:** 10.1002/jcp.25685

**Published:** 2016-11-30

**Authors:** Teresa Gamucci, Lucia Mentuccia, Clara Natoli, Isabella Sperduti, Alessandra Cassano, Andrea Michelotti, Luigi Di Lauro, Domenico Sergi, Alessandra Fabi, Maria G. Sarobba, Paolo Marchetti, Maddalena Barba, Emanuela Magnolfi, Marcello Maugeri‐Saccà, Ernesto Rossi, Valentina Sini, Antonino Grassadonia, Domenica Pellegrini, Antonino Astone, Cecilia Nisticò, Franco Angelini, Angela Vaccaro, Arianna Pellegrino, Claudia De Angelis, Michela Palleschi, Luca Moscetti, Ilaria Bertolini, Simonetta Buglioni, Antonio Giordano, Laura Pizzuti, Patrizia Vici

**Affiliations:** ^1^ Medical Oncology Unit Frosinone Italy; ^2^ Department of Medical, Oral and Biotechnological Sciences Centro Scienze dell’ Invecchiamento e Medicina Traslazionale ‐ CeSI‐MeT Chieti Italy; ^3^ Bio‐Statistics Unit, Regina Elena National Cancer Institute Rome Italy; ^4^ Department of Medical Oncology Policlinico Universitario A. Gemelli Rome Italy; ^5^ Oncology Unit I Azienda Ospedaliera Universitaria Pisana Pisa Italy; ^6^ Division of Medical Oncology 2 Regina Elena National Cancer Institute Rome Italy; ^7^ Division of Medical Oncology 1 Regina Elena National Cancer Institute Rome Italy; ^8^ Medical Oncology Unit Nuoro Italy; ^9^ Department of Clinical and Molecular Medicine “Sapienza” University of Rome Azienda Ospedaliera Sant'Andrea Rome Italy; ^10^ Scientific Direction Regina Elena National Cancer Institute Rome Italy; ^11^ Medical Oncology Unit Regina Apostolorum Hospital Albano, Rome Italy; ^12^ Medical Oncology Unit, Ospedale San Pietro Fatebenefratelli Rome Italy; ^13^ Medical Oncology Unit, Policlinico Umberto I Rome Italy; ^14^ Medical Oncology Unit, Belcolle Hospital Viterbo Italy; ^15^ Department of Medical and Surgical Sciences for Children and Adults Azienda Ospedaliero‐Universitaria Policlinico di Modena Modena Italy; ^16^ Department of Pathology Regina Elena National Cancer Institute Rome Italy; ^17^ Sbarro Institute for Cancer Research and Molecular Medicine and Center for Biotechnology College of Science and Technology Temple University Philadelphia Pennsylvania

## Abstract

Bevacizumab in combination with taxanes in HER2‐negative metastatic breast cancer (MBC) patients has shown improved progression‐free survival (PFS), despite the lack of clear overall survival (OS) benefit. We performed a retrospective analysis to evaluate the impact of paclitaxel‐bevacizumab and of maintenance therapy with bevacizumab (BM) and endocrine therapy (ET) in the real‐world practice. We identified 314 HER2‐negative MBC patients treated in 12 cancer centers. Overall, the median PFS and OS were 14 and 40 months, respectively. Among the 254 patients potentially eligible for BM, 183 received BM after paclitaxel discontinuation until progression/toxicity. PFS and OS were improved in patients who had received BM in comparison with those potentially eligible but who did not receive BM (*P*< 0.0001 and *P* = 0.001, respectively). Results were confirmed when adjusting for propensity score. Among the 216 hormone‐receptor positive patients eligible for BM, a more favorable PFS and OS were observed when maintenance ET was administered (*P *< 0.0001). Multivariate analysis showed that PS, BM, number of disease sites and maintenance ET were related to PFS, while response and maintenance ET were related to OS. In hormone‐receptor positive patients, BM produced a significant PFS and a trend towards OS benefit only in absence of maintenance ET (*P* = 0.0007 and *P* = 0.06, respectively). In the triple‐negative subgroup, we observed a trend towards a better OS for patients who received BM (*P* = 0.06), without differences in PFS (*P* = 0.21). Our results confirmed the efficacy of first‐line paclitaxel‐bevacizumab in real‐world practice; both BM and maintenance ET significantly improved PFS and OS compared to no maintenance therapies. J. Cell. Physiol. 232: 1571–1578, 2017. © 2016 Wiley Periodicals, Inc.

Metastatic breast cancer (MBC) is still considered incurable, with a median survival of 2–4 years (Reddy et al., 2012). In the HER2‐positive disease, HER2 blocking agents in combination with chemotherapy are the mainstay of treatment, and endocrine therapy is the preferred first‐line choice in patients with tumors expressing hormonal receptors. Conversely, there is no standard treatment for advanced HER2‐negative tumors not expressing hormonal receptors or for tumors resistant to ET. In these patients, novel treatment combinations and potential targets are urgently needed (Gogineni and DeMichele, 2012).

Angiogenesis plays an essential role in tumor growth and progression. Vascular endothelial growth factors (VEGF) regulates tumor angiogenesis by stimulating endothelial cell proliferation and migration, inhibiting apoptosis, remodelling extracellular matrix, and increasing vascular permeability. Bevacizumab is a humanized monoclonal antibody directed against VEGF‐A. It prevents binding of VEGF to receptors on endothelial cells, leading to inhibition of angiogenesis and tumor growth, promoting blood vessels degradation, potentiating the effect of chemotherapy (Ferrara et al., 2004). In the first‐line setting, three randomized trials (E2100, AVADO, RIBBON I) showed progression free survival (PFS) and response rate (RR) advantage when combining BM with chemotherapy against chemotherapy alone, without advantage in overall survival (OS) (Miller et al., 2007; Miles et al., 2010; Robert et al., 2011). Moreover, the ATHENA trial evaluated BM toxicity and, secondarily, efficacy, in combination with taxane‐based chemotherapy, in a large real‐world patient cohort. The evidence observed was consistent with results from previous trials, with patients who continued BM after chemotherapy discontinuation showing more favorable outcomes (Smith et al., 2011b). Increasing evidence indicates improved efficacy of continued antiangiogenetic therapy in BC, with data deriving both from the preclinical and clinical settings. Indeed, tumor vessels may rapidly re‐grow after BM discontinuation (Mancuso et al., 2006; Ebos et al., 2009; Pàez‐Ribes et al., 2009). Recently, in the TANIA trial, continuation of bevacizumab‐based treatment beyond progression has been associated with improved PFS. In addition, results from the IMELDA trial have confirmed the efficacy of maintenance therapy with capecitabine plus BMversus BM alone after a first line treatment with docetaxel‐bevacizumab (Gligorov et al., 2014; von Minckwitz et al., 2014).

Notwithstanding the lack of clear OS benefit, randomized trials and several metanalyses confirmed the advantage of BM in RR and PFS for patients with MBC (Valachis et al., 2010; Rossari et al., 2012; Miles et al., 2013; Kümler et al., 2014; Fang et al., 2015; Li et al., 2015), and its use in combination with taxanes is considered a promising strategy in HER2‐negative MBC.

To help fill the gap of knowledge between clinical trials and actual clinical practice, we performed a multicenter retrospective observational study of first‐line bevacizumab‐paclitaxel and maintenance therapy in HER2‐negative MBC patients.

## Patients and Methods

The primary objective of the study was to assess the efficacy of BM combined with paclitaxel as first‐line treatment for HER2‐negative MBC. Secondarily, we evaluated the impact on outcomes of bevacizumab maintenance (BM), alone or in combination with maintenance ET in the subset of patients with hormone‐receptor positive tumors.

HER2‐negative MBC patients not enrolled in clinical trials were retrospectively and sequentially identified and recruited from 12 Italian cancer centers. Treatment schedules were paclitaxel administered weekly, at the dose of 80 or 90 mg/m^2^, plus bevacizumab, 10 mg/kg every 2 weeks or 15 mg/kg every 3 weeks (depending on the physician preference), with or without BM after paclitaxel discontinuation. Treatment was continued until disease progression, unacceptable toxicity, or refusal. Endocrine sequential maintenance treatment was given based on physician choice and according to standard guidelines after paclitaxel discontinuation in patients with hormone‐receptor positive tumors.

Treatment efficacy was evaluated by conventional Response Evaluation Criteria in Solid Tumors (RECIST) criteria. All the patients signed a written informed consent, and the institutional ethic committees approved the retrospective analysis.

## Statistical Analysis

Descriptive statistics were used to summarize patient characteristics.

The associations between variables were tested by chi–square or Fisher's exact test. Survival estimates were computed and compared by Kaplan–Meier product‐limit and log–rank test. The median follow‐up was estimated with the Kaplan–Meier reverse method.

To minimize the differences in patients’ covariates, which could become confounding factors in the examination of treatment effects in a non‐randomized cohort, we used the propensity score match to create groups of patients who were similarly likely to receive a given treatment on the basis of their baseline characteristics (D'Agostino et al., 1998).

A multivariate Cox proportional hazard model was developed in the hormone‐receptor positive subgroup using stepwise regression (forward selection). Variables testing significant at the univariate analysis were entered into the model, which also included interaction terms. Enter limit and remove limit were *P* = 0.10 and *P* = 0.15, respectively. Potential markers of prognostic significance included: age, ECOG PS, tumor histology, tumor size, molecular subtype, type of surgery, adjuvant and advanced treatments, type and number of metastatic sites, BM, maintenance ET, and treatment response.

When considering BM, we included in the analysis only patients who had received adequate paclitaxel treatment and who had not experienced disease progression. To determine the role of maintenance ET, we comprised patients with hormone‐receptor positive tumors only. SPSS software (SPSS version 21.0, SPSS Inc., Chicago, IL) was used for all statistical evaluations.

## Results

From January 2008 to February 2015, 314 MBC patients were retrospectively identified and enrolled at 12 Italian cancer centres. Included patients had received at least one paclitaxel‐bevacizumab cycle. Main patient and tumor characteristics are listed in Table 1. Median age was 55 years, with 21.6% of patients being older than 65 years. Median ECOG PS status was 0. At baseline, immunostaining of surgical specimens showed 80.9% ER and/or PgR positive tumors, 71.3% ER and PgR positive and 15.9% triple‐negative cancers. Three patients showed HER2‐positivity on initial tumor, but metastatic biopsy was HER2‐negative. The majority of the patients had visceral metastasis (61.7%), bone exclusive disease was recorded in 41 patients (13.1%), and 193 patients (61.5%) had multiple metastatic sites.

**Table 1:** Main patient and tumors characteristics in the overall population

Main baseline patient characteristics	n (%)
Age, median (range)	55 (27–82)
ECOG PS	
0	210 (67)
1–2	104 (33)
Histology	
Ductal	275 (87.6)
Lobular	29 (9.2)
Other	10 (3.2)
Hormone receptor and HER‐2 status at initial diagnosis	
ER and/or PgR positive	254 (80.9)
Triple negative	50 (15.9)
HER2 positive	3 (1.0)
Unknown	7 (2.2)
KI67	
>14%	211 (67.2)
≤14%	103 (32.8)
Neoadjuvant /Adjuvant treatment	
Neoadjuvant chemotherapy	78 (24.8)
Adjuvant chemotherapy	181 (57.6)
Adjuvant taxanes	104 (33.1)
Adjuvant endocrine therapy	211 (67.2)
Adjuvant radiotherapy	161 (51.3)
Metastatic at diagnosis	
Yes	55 (17.5)
No	259 (82.5)

ECOG PS, eastern cooperative oncology group performance status; ER, estrogen receptor; PgR, progesterone receptor; n, number.

### Treatment received

Overall, 24.8% of patients had received neoadjuvant chemotherapy and 57.6% adjuvant chemotherapy. Two hundred and eleven patients had received adjuvant ET (67.2% of the overall population, 92% of the hormone‐receptor positive tumors). The remaining 8% of hormone‐receptor positive patients had not received adjuvant ET due to physician decision.

Among the 254 patients with hormone‐receptor positive tumors, 39% had received one or more ET for advanced disease (median 1, range 1–2), prior to first‐line paclitaxel‐bevacizumab.

Overall, the median duration of treatment with paclitaxel and BM was 8 months (range, 1–41). After paclitaxel discontinuation, BM was not administered to 60 patients because of disease progression during (27 patients) or at the end of chemotherapy (25 patients), or due to toxicity (8 patients). These patients were not included in the prognostic factors analysis. Further 71 patients did not receive BM due to physician or patient decision, even in absence of progression or toxicity. Overall, 183 patients (58.3%) received BM after paclitaxel discontinuation. Main patient and tumor characteristics according to BM administration are listed in Supplementary Table S1. Among the 254 patients with hormone‐receptor positive tumors, 70.5% received ET as maintenance treatment after paclitaxel discontinuation, which was combined with BM in 51.2% of patients, and administered without BM in 19.3% of patients (Supplementary Table S2).

### Efficacy

All 314 patients were evaluable for response. We observed 31 complete responses (CR) (9.9%) and 177 partial responses (PR) (56.4%), for an overall RR of 66.2% (95%CI, 61.0–71.5). Stable disease (SD) was observed in 80 patients (25.5%). No significant differences in RR were observed by molecular subtype (hormone‐receptor positive or triple negative tumors, *P* = 0.13). Disease control rate, defined as CR, PR, and SD lasting ≥6 months, was recorded in 87.3% of patients, with significant differences between hormone‐receptor positive and triple‐negative tumors (90.2% vs. 74.0%, *P* = 0.002) (Table 2). Objective responses did not differ by disease sites, adjuvant taxanes, paclitaxel, or BM doses/schedules.

**Table 2:** Response and disease control rate according to molecular subtype

Molecular Subtype	n	CR/PR, n (%)	*P* value	DCR, n (%)	*P* value
Triple negative	50	29 (58.0)	0.13	37 (74.0)	0.002
ER and/or PgR positive	254	175 (69.0)		229 (90.2)	

CR, complete response; PR, partial response; DCR, disease control rate; ER, estrogen receptor; PgR, progesterone receptor; n, number.

**Figure 1:**
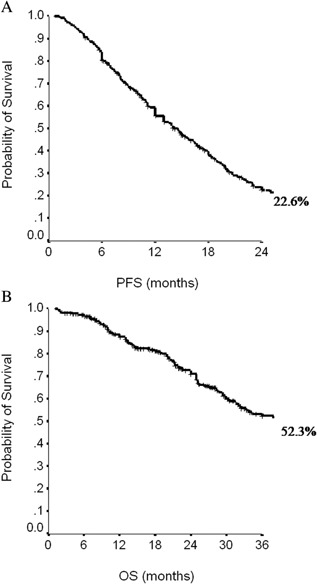
Progression‐free survival (PFS, A) and overall survival (OS, B) in the overall population.

In the overall patient population, at a median follow up of 27 months (95%CI, 25–30), median PFS was 14 months (95%CI, 12–16), and median OS was 40 months (95%CI, 33–47) (Fig. 1a and b). The median PFS in the triple‐negative subgroup was 9 months (95%CI, 6–12), whereas in the subset of patients with hormone‐receptor positive tumors median PFS was 16 months (95%CI, 14–18) (*P* = 0.001). The median OS was 25 months (95%CI, 17–33) in the triple‐negative subgroup, while it was 41 months (95%CI, 32–50) in patients with hormone‐receptor positive tumors (*P* = 0.009). Among the 179 (70.5%) hormone‐receptor positive patients who received maintenance ET, median PFS and median OS were 18.5 (95%IC, 16.5–20.4) and 59 (95%CI, 40.6–77.3) months, respectively. Conversely, among the 75 (29.5%) hormone‐receptor positive patients who did not received maintenance ET, median PFS and median OS were 8.1 (95%CI, 6.3–9.9) and 25 (95%CI, 20.6–29.4) months, respectively. Therefore, maintenance ET after paclitaxel‐bevacizumab offered a significant benefit both in PFS and OS (*P* < 0.0001).

**Figure 2:**
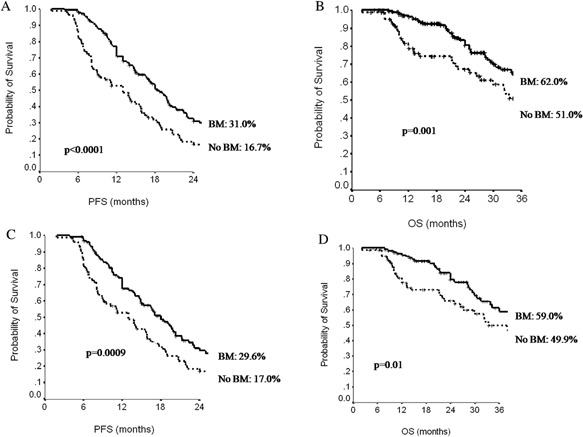
Progression‐free survival (PFS, A) and overall survival (OS, B) according to administration of bevacizumab maintenance (BM) and adjusted for propensity score (PFS, C; OS, D).

Among the 254 patients potentially eligible for BM, median PFS of 183 patients who received BM was 18 months (95%CI, 17–20) compared with 13 (95%CI, 10–16) months for patients who did not receive BM (*P *< 0.0001) (Fig. 2a). Similarly, the median OS were 55 (95%CI, 36–74) and 38 months (95%CI, 28–47) (*P* = 0.001), respectively (Fig. 2b). These data were confirmed when adjusting for propensity score (Fig. 2c and d).

**Figure 3:**
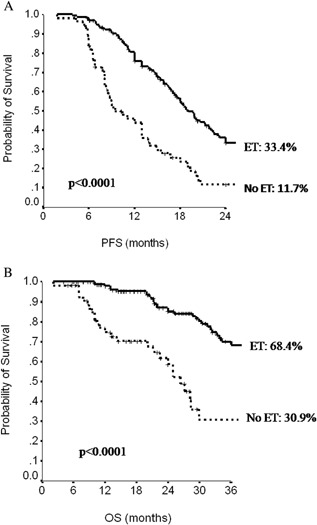
Progression‐free survival (PFS, A) and overall survival (OS,B) in hormone‐receptor positive patients according administration of hormonal therapy (ET) at the end of first‐line chemotherapy.

Figure 3 shows PFS and OS curves in the subset of 216 hormone‐receptor positive patients also eligible for BM. Patients receiving maintenance ET had more favorable outcomes, independently on BM, (median PFS: ET 19 months, no‐ET 9 months, *P *< 0.0001; median OS: ET 64 months, no‐ET 26 months, *P*< 0.0001). Maintenance ET offered a significant benefit in the median PFS and OS both in ET naive patients and in those who had previously received ET.

When exploring BM in hormone‐receptor positive patients, at the multivariate analysis, PS, BM, number of disease sites and maintenance ET were related to PFS, while treatment response and maintenance ET were associated with OS (Table 3). Results were confirmed when adjusting for propensity score (data available upon request). In this same subset, BM, in the absence of maintenance ET, produced a significant advantage in the median PFS, from 8 to 13 months (*P* = 0.0007). These results were confirmed also in patients with an adequate chemotherapy length (at least 18 paclitaxel administration). Conversely, in patients having received maintenance ET, the administration of BM was associated with a trend in median PFS benefit (20 vs. 16 months, *P* = 0.07). However, in patients treated with BM for more than 2 months, the advantage of adding BM became significant, even in presence of maintenance ET (*P* = 0.04). In patients with an adequate length of chemotherapy and maintenance ET, the advantage in the median PFS of adding BM was lost (*P* = 0.20), even if the duration of BM was adequate.

**Table 3:** Multivariate analysis with Cox Regression model on hormone‐receptor positive patients for PFS and OS

	PFS		OS	
Variables	HR (95%CI)	*P*	HR (95%CI)	*P*
ECOG PS (1/2 vs. 0)	1.39 (1.0–1.94)	0.05	—	ns
Number of disease site (>1 vs. 1)	1.44 (1.01–2.05)	0.04	—	ns
Bevacizumab maintenance (no vs. yes)	1.52 (1.08–2.14)	0.02	—	ns
Maintenance endocrine therapy (no vs. yes)	2.45 (1.71–3.51)	< 0.0001	4.69 (2.84–7.74)	< 0.0001
Treatment response (no vs. yes)	—	—	1.79 (1.08–2.97)	0.03

PFS, progression‐free survival; OS, overall survival; ECOS PS, eastern cooperative oncology group performance status; HR, hazard ratio.

In the subset of hormone‐receptor positive patients not receiving maintenance ET, the administration of BM prolonged median OS from 22 to 28 months (*P* = 0.06). Conversely, no advantage from BM was observed in patients having received maintenance ET (*P* = 0.89).

**Figure 4:**
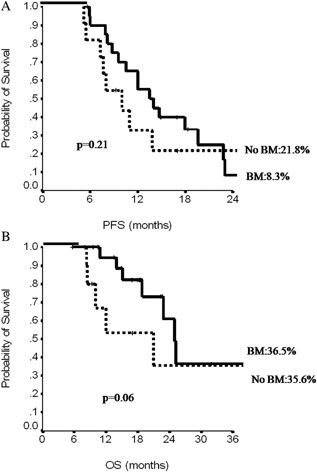
Progression‐free survival (PFS, A) and overall survival (OS, B) in triple‐negative patients according to administration of bevacizumab maintenance (BM).

Only 34 out of the 50 triple‐negative patients were amenable to BM. We further excluded two patients because of a change in the HER2 status. Among the remaining 32 patients, 21 received BM after paclitaxel discontinuation, while 11 did not. There was no significant difference in the median PFS by BM administration (*P* = 0.21) (Fig. 4a), whereas a trend towards a better median OS was observed for patients who received BM (25 vs. 21 months, *P* = 0.06) (Fig. 4b). Among the 21 triple‐negative patients who had received BM, 14 received subsequent chemotherapy lines (median: 3 lines). Among the 11 triple‐negative patients who did not received BM, only 5 received subsequent chemotherapy lines (median: 1 more line).

## Discussion

In the first‐line setting, the E2100 trial demonstrated significant PFS and RR improvements with paclitaxel‐bevacizumab compared with paclitaxel alone in patients with HER2‐negative MBC (Miller et al., 2007). Subsequently, the AVADO trial, evaluating bevacizumab‐docetaxel as first‐line therapy (Miles et al., 2010), showed a small improvement in median PFS compared with the control arm, and RR was also increased in the experimental arm. In the RIBBON‐1 trial, evaluating the combination of BM with capecitabine, taxane‐based, or anthracycline‐based chemotherapy as first‐line treatment for HER2‐negative MBC, median PFS, and RR were more favorable for any bevacizumab‐chemotherapy combinations compared with placebo‐arms, and PFS was longer in the capecitabine cohort (*P* = 0.0002) (Robert et al., 2011). However, no significant difference in OS were observed. The ATHENA trial evaluated BM combined with taxane‐based chemotherapy in a large patient cohort from the real‐world practice. The median time to progression (TTP) was 9.5 months, RR was 52%, SD was 33% (Smith et al., 2011b) and the median OS was 25.2 months (Smith et al., 2011a).

In triple‐negative tumors there is no established standard therapy, and taxane‐based regimens represent a reasonable approach. A meta‐analysis of triple‐negative MBC patients from the E2100, AVADO, and RIBBON‐1 trials showed a significant improvement in PFS for patients treated with chemotherapy plus BM (8.1 vs. 5.4 months, *P* = 0.0002), RR was significantly higher with bevacizumab‐containing therapy than with chemotherapy alone (42% vs. 23%, *P *< 0.0001), median OS was 18.9 versus 17.5 months, and 1‐year OS rates were 71% versus 65%. (Miles et al., 2013). An exploratory subgroup analysis of triple‐negative BC patients from the ATHENA study was confirmative, with a median TTP of 7.2 months and a median OS of 18.3 months, in comparison to 9.5 and 25.2 months, respectively, in the overall population (Thomssen et al., 2012).

An exploratory analysis of data from the subpopulation of taxane‐pretreated patients in the E2100 and AVADO trials, confirmed the benefit of adding BM even in this patient population Miles et al., 2010). This result is fully consistent with our findings. A metanalysis of first‐line phase III trials (Rossari et al., 2012) and a pooled and subgroup analysis of individual patients data (Miles et al., 2013) confirmed the advantage in RR and PFS without OS benefit, except for 1‐year survival rate, showing 5 months advantage in favor of the BM arm (Miles et al., 2013).

The lack of clear survival benefit may be partly due to an inadequate length of BM administration. Preclinical data indicate that tumor vessels re‐growth after BM discontinuation, thus suggesting the need of prolonged administration until, and, possibly, beyond progression (Mancuso et al., 2006; Dirix et al., 2010; Ebos and Kerbel, 2011; Bennouna et al., 2013). Further, explanations may be the limited power of the studies, treatment crossover, and confounding effect of post‐progression treatments.

The lack of survival benefit has made the use of BM controversial, and it is crucial to define which magnitude of benefit may be expected in real‐world practice, outside clinical trials. Recently, a real‐world experience of ESME database of 2,127 HER2‐negative patients having received as first‐line treatment paclitaxel‐bevacizumab and 1,299 patients having received only paclitaxel, has showed a significant benefit in OS for the combination arm (HR 0.67). Median PFS was 8.1 months and 6.4 months in the combination group and chemotherapy alone arm, respectively (HR 0.74). Results were confirmed in subgroup analysis by molecular subtype (Delaloge et al., 2016).

The continuation of BM was investigated in a retrospective analysis of patients treated with paclitaxel‐bevacizumab in first or subsequent lines, which showed significant better PFS (13.7 vs.5.4 months, *P* < 0.001) and OS (37.4 vs. 13.9 months, *P* = 0.002) in patients having received BM (Redondo et al., 2014). In a subgroup analysis of the ATHENA trial, BM, in comparison with no BM, offered a longer PSF (11.6 vs. 6.7 months) and OS (30 vs. 18.4 months) (Smith et al., 2011a). Results from the TANIA phase III study (von Minckwitz et al., 2014) support the validity of a beyond progression continued VEGF inhibition with further BM.

The concomitant targeting of VEGF and estrogen pathways may provide enhanced benefit in patients with hormone‐receptor positive BC, with preclinical and clinical data supporting the hypothesis of a synergistic action (Bottini et al., 2006; Qu et al., 2008; Suriano et al., 2008; Banerjee et al., 2010; Forero‐Torres et al., 2010; Traina et al., 2010; Yardley et al., 2011). A phase II randomized trial (CALGB 40503) tested as first‐line treatment letrozole versus letrozole‐bevacizumab, showing advantage in PFS (15.6 vs. 20.2 months, *P* = 0.016), and no OS benefit, with an increase in serious adverse event in the experimental arm (Dickler et al., 2015).

The continuation of BM and the addition of ET following first‐line therapy was investigated in the retrospective analysis including 40 patients with estrogen receptor‐positive tumors from (Redondo et al., 2014). Median PFS was 21.9 months in the group receiving BM and ET maintenance therapy, compared with 10.6 months of those receiving only BM (*P* = 0.065), and median OS was not reached in the arm with maintenance ET (Redondo et al., 2014). The GINECO phase III study randomized HER2 negative, estrogen‐receptor positive patients who had not progressed after first‐line treatment with paclitaxel‐bevacizumab, to continuation of paclitaxel‐bevacizumab or cross to BM plus exemestane. The trial failed to show superior efficacy of ET maintenance compared with continuation of chemotherapy (Trédan et al., 2016).

The efficacy and safety of adding a third agent (cytotoxic or biologic) to BM/taxane regimen was evaluated in a recent meta‐analysis including seven randomized trials and 1,124 HER2‐negative advanced breast cancer patients treated in first‐line setting (Liu et al., 2016). Data showed a statistically significant advantage in overall RR but not in PFS and OS, with an increase in toxicity.

In the present study, our findings on the combination of paclitaxel‐bevacizumab administered as first‐line treatment in real‐world practice fairly compares with the results of the ATHENA trial, showing a RR of 66.2%, a median PFS of 14 months, and a median OS of 40 months, whereas in the ATHENA trial these estimates were 52%, 9.5 and 25 months, respectively (Smith et al., 2011a,b). Differences in terms of visceral involvement (61.8% vs. 85.1%) and number of triple‐negative tumors (15.9% vs. 26.5%) may have favorably influenced our results. In our study, the median PFS and OS were significantly higher in hormone‐receptor positive tumors than in the triple‐negative subtype (16 vs. 9 months, *P* = 0.001, and 41 vs. 25 months, *P* = 0.009). However, maintenance ET in the subset of hormone‐receptor positive patients certainly affected OS. Indeed, am ong the hormone‐receptor positive patients who received maintenance ET, the median PFS and OS were significantly more favorable than in patients not treated with maintenance ET (*P *< 0.0001). Moreover, in our patient population, previous ET in adjuvant/advanced setting did not decrease the efficacy of subsequent maintenance ET.

In the subset of 216 hormone‐receptor positive patients eligible for BM, maintenance ET offered a clear advantage both in median PFS (19 vs. 9 months, *P *< 0.0001) and in median OS (64 vs. 26 months, *P *< 0.0001), compared with patients with no maintenance ET, independently on the administration of BM.

In the present study, overall, the administration of BM prolonged median PFS from 13 to 18 months (*P *< 0.0001), and median OS from 38 to 55 months (*P* = 0.001), confirming the ATHENA results, where median TTP was prolonged from 9.5 to 11.6 months and median OS rose from 25 to 30 months (Smith et al., 2011a). The administration of BM, in absence of maintenance ET, in the subset of hormone‐receptor positive patients offered a clear advantage in median PFS. Conversely, in patients receiving both maintenance therapies, the advantage of BM was lost, and eventually regained when the length of BM was adequate (> 2 months) (*P* = 0.04). These latter data seem to support the need of prolonging BM administration. In patients receiving maintenance ET and having received an adequate chemotherapy duration, no advantage in PFS from BM was observed (*P* = 0.20), but numbers are small.

In the hormone‐receptor positive subgroup the administration of BM, in absence of maintenance ET, prolonged OS (*P* = 0.06), and this trend disappeared when concomitant maintenance ET was delivered (*P* = 0.89).

These results confirmed the value of an adequate chemotherapy duration, followed by adequate BM and maintenance ET in hormone‐receptor positive patients.

In the small subset of triple‐negative MBC patients, median PFS and OS were 9 and 25 months, respectively, comparing favorably with the ATHENA results where these estimates were 7.2 and 18 months (Thomssen et al., 2012). In our triple‐negative patients, BM did not offer a PFS advantage (*P* = 0.21), while a trend toward OS benefit was observed (*P* = 0.06). A possible explanation deriving from our data may be that the prolonged administration of BM allowed an increased number of post‐progression treatment lines, and possibly favored their efficacy due to the sustained anti‐angiogenetic effect.

Our results are consistent with previous observational studies, and confirmed the advantage of adding BM to a first‐line chemotherapy with paclitaxel‐bevacizumab in HER2‐negative MBC patients (Smith et al., 2011a; Redondo et al., 2014; von Minckwitz et al., 2014). Our data also support the relevant impact of an adequate length of chemotherapy and prolonged BM. In patients not receiving maintenance ET, the importance of an adequate BM is clear, offering advantage in both PFS and OS, and confirming the importance of sustained VEGF inhibition for long‐term disease control.

Results from our subset of triple‐negative patients are intriguing, given the suggestion for longer OS in patients under BM, even if there was no impact on PFS. Although our numbers invite caution, the prolonged administration of BM may have favored post‐progression treatments and impacted OS.

Our results confirmed the relevance of adding maintenance ET in hormone‐receptor positive patients. Such relevance seems to exceed the favorable effect of BM in this subgroup of patients.

The present work has important limitations, which mainly stem from its retrospective observational nature which makes it prone to confounding and bias. Moreover, our patient population is heterogeneous, several doses and schedules of treatment were used, and the sample size is relatively small when considering subgroups. The existence of possible major differences between patients receiving maintenance therapies versus remaining patients must be taken into account. In our study, the two groups analyzed showed significant differences in terms of PS. To minimize the PS‐related selection bias, survival analysis were adjusted using propensity score. However, case matching by PS could not remove other potentially important causes of bias from unknown confounders possibly including prior/subsequent therapies, comorbidities, and differences in disease biology. In these regards, it is worth mentioning that data concerning co‐morbidities and safety were not available for analysis purposes. However, our study also has important strengths. Among them, the suggestion of the efficacy of both BM and maintenance ET, and the statistical methodology applied, with the use of propensity score to reduce the selection bias. Moreover, data deriving from real‐world studies provide a realistic picture of what really takes place outside clinical trials.

In conclusion, our results confirm that paclitaxel‐BM represent one of among the effective therapeutic strategies in real‐world patients with HER2‐negative MBC. In the absence of disease progression or significant toxicity, the continuation of BM, and the addition of ET in the hormone‐receptor positive subgroup, appear to be a reasonable approach for more favorable outcomes.

## Authors’ Contributions

TG and PV contributed to study conception, critical revision of the manuscript for important contents. PV and LP contributed to manuscript drafting, study conduct, data collection, and analysis planning. PM, MB, MMS, SB, and AG critically revised the manuscript for important intellectual content. IS contributed to the statistical analysis. LM, CN, AC, AM, LDL, DS, AF, MGS, EM, ER, VS, AG, DP, AA, CN, FA, AV, AP, CDA, MP, LM, and IB contributed to the data collection, database set up and implementation, contribution to data analysis. All authors read and approved the final version of this manuscript and are responsible for all the aspects of the work.

## Supporting information

Additional supporting information may be found in the online version of this article at the publisher's web‐site.

Supplementary Table S1.

Supplementary Table S2.
