# Supplementary material for: A Real‐World Multicentre Retrospective Study of Paclitaxel‐Bevacizumab and Maintenance Therapy as First‐Line for HER2‐Negative Metastatic Breast Cancer
Source: J Cell Physiol. 2016 Nov 30;232(6):1571–8. doi: 10.1002/jcp.25685 (PMC6220933; doi:10.1002/jcp.25685)
Supplement: Supplementary file 1 — Supplementary Table S1. [file JCP-232-1571-s001.doc]

**Supplementary table 1.** Main patient and tumors characteristics according to BM administration (254 pts)

| **Main baseline patient characteristics** | **BM (183 pts)**  **n(%)** | **no BM (71)**  **n(%)** |
| --- | --- | --- |
| *Age, Median (range)* | 54 (30-79) | 53 (27-82) |
| *ECOG PS*  0  1-2 | 131 (71.6)  52 (28.4) | 40 (56.3)  31 (43.7) |
| *Histology*  Ductal  Lobular  Other | 162 (88.5)  16 (8.8)  5 (2.7) | 61 (86.0)  5 (7.0)  5 (5.6) |
| *Hormone receptor and HER-2 status at initial diagnosis*  ER and/or PgR positive  Triple negative  Unknown | 157 (85.8)  22 (12.0)  6 (2.2) | 59 (83.1)  10 (14.1)  2 (2.8) |
| *KI67*  >14%  ≤14%  Unknown | 115 (62.8)  51 (27.9  17 (9.3) | 47 (66.2)  13 (18.3)  11(15.5) |
| *Neoadjuvant /Adjuvant treatment*  Neoadjuvant chemotherapy  Adjuvant chemotherapy  Adjuvant taxanes  Adjuvant endocrine therapy  Adjuvant radiotherapy | 32 (17.5)  107 (58.5)  61 (33.3)  133 (72.7)  97 (53.0) | 12 (16.9)  37 (52.1)  14 (19.7)  42 (59.2)  35 (49.3) |
| *Metastatic at diagnosis*  Yes  No | 28 (15.3)  155 (84.7) | 18 (25.4)  53 (74.6) |

Abbreviations: BM, bevacizumab maintenance; pts, patients; ECOG PS, Eastern Cooperative Oncology Group Performance Status; ER, Estrogen Receptor; PgR, Progesterone Receptor; n, number.
