# Supplementary material for: A Real‐World Multicentre Retrospective Study of Paclitaxel‐Bevacizumab and Maintenance Therapy as First‐Line for HER2‐Negative Metastatic Breast Cancer
Source: J Cell Physiol. 2016 Nov 30;232(6):1571–8. doi: 10.1002/jcp.25685 (PMC6220933; doi:10.1002/jcp.25685)
Supplement: Supplementary file 2 — Supplementary Table S2. [file JCP-232-1571-s002.doc]

**Supplementary table 2.** Main patient and tumors characteristics according to maintenance ET administration in ER and/or PgR positive patients (213 pts)

| **Main baseline patient characteristics** | **ET (162 pts)**  **n(%)** | **no ET (51 pts)**  **n(%)** |
| --- | --- | --- |
| *Age, Median (range)* | 54 (30-79) | 53 (27-82) |
| *ECOG PS*  0  1-2 | 112 (69.1)  50 (30.9) | 32 (62.7)  19 (37.3) |
| *Histology*  Ductal  Lobular  Other | 143 (88.3)  15 (9.3)  4 (2.5) | 43 (84.4)  4(7.8)  4(7.8) |
| *Ki67*  >14%  ≤14%  Unknown | 99 (61.1)  48 (29.6)  15 (9.3) | 36 (70.6)  11 (21.6)  4 (7.8) |
| *Neoadjuvant /Adjuvant treatment*  Neoadjuvant chemotherapy  Adjuvant chemotherapy  Adjuvant taxanes  Adjuvant endocrine therapy  Adjuvant radiotherapy | 22 (13.6)  86 (53.1)  38 (23.5)  130 (80.2)  83 (51.2) | 9 (17.6)  32 (62.7)  21 (41.2)  40 (78.4)  25 (49.0) |
| *Metastatic at diagnosis*  Yes  No | 34 (21.0)  128 (79.0) | 8 (15.7)  43 (84.3) |

Abbreviations: ET, endocrine treatment; Pts, patients ; ECOG PS, Eastern Cooperative Oncology Group Performance Status; ER, Estrogen Receptor; PgR, Progesterone Receptor; n, number.
